# Supplementary figures and images for: Cyanobacterial contribution to the genomes of the plastid-lacking protists
Source: BMC Evol Biol. 2009 Aug 11;9:197. doi: 10.1186/1471-2148-9-197 (PMC3087521; doi:10.1186/1471-2148-9-197)

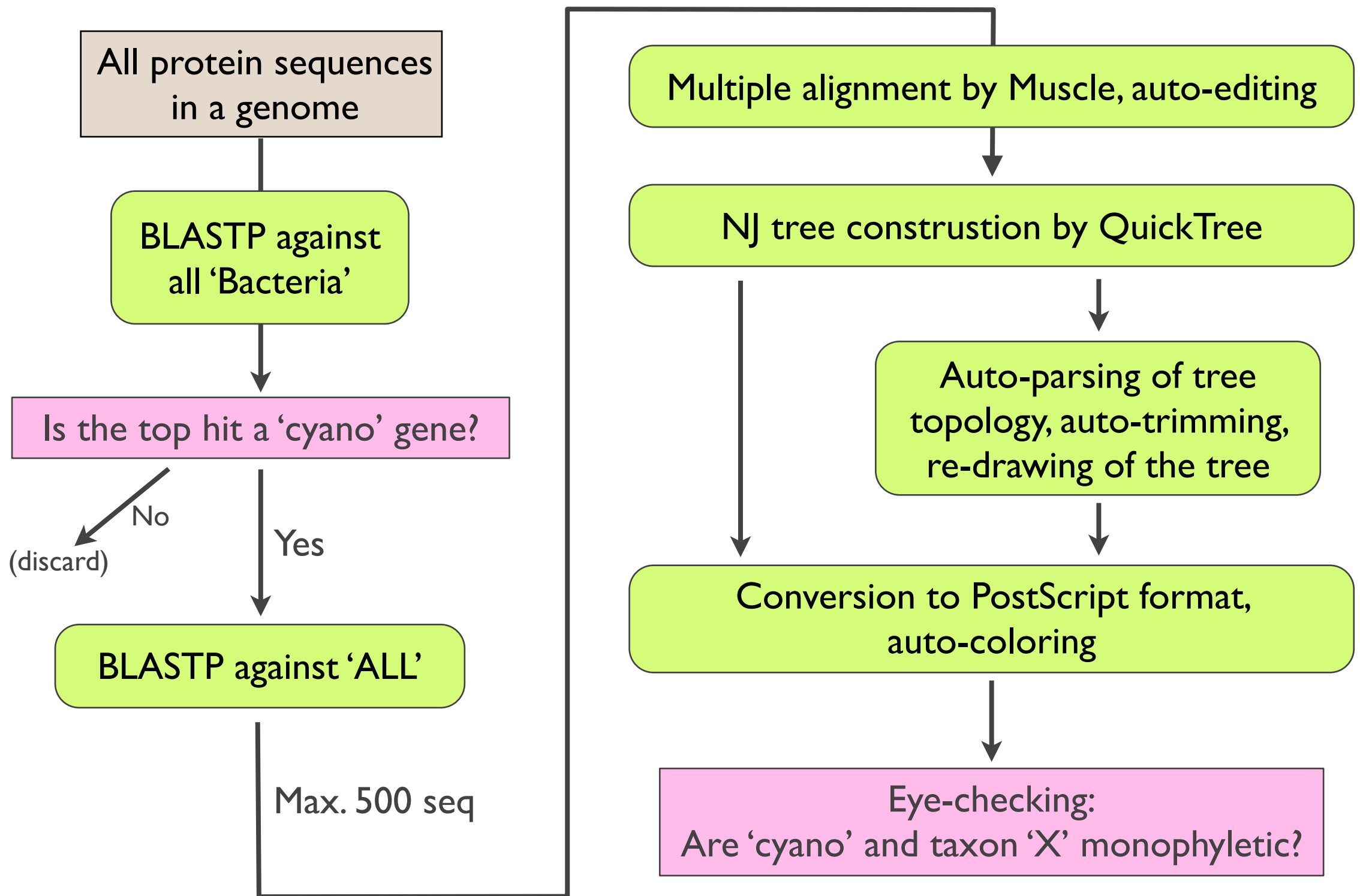

Add\_file 1: Fig. 7 Flow chart of procedures in the phylogenetic analysis

Supplement: Additional file 1 — Supplemental Figure 7. Flow chart of procedures used in the phylogenetic analyses. [file 1471-2148-9-197-S1.pdf]

A Bayesian tree  
BI/ML

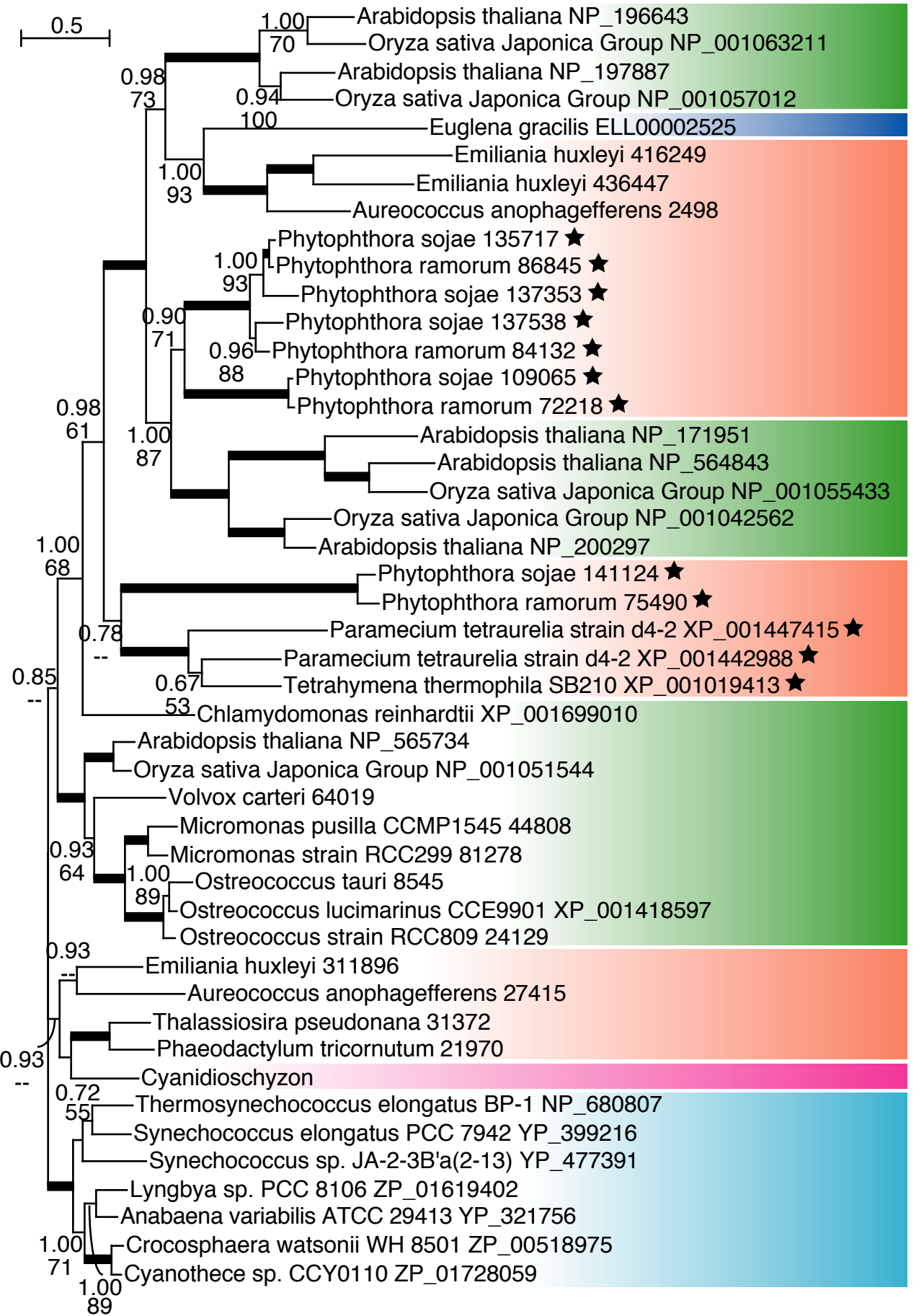

Supplement: Additional file 2 — Supplemental Figure 8. MrBayes consensus tree of folate/biopterin transporter genes. [file 1471-2148-9-197-S2.pdf]

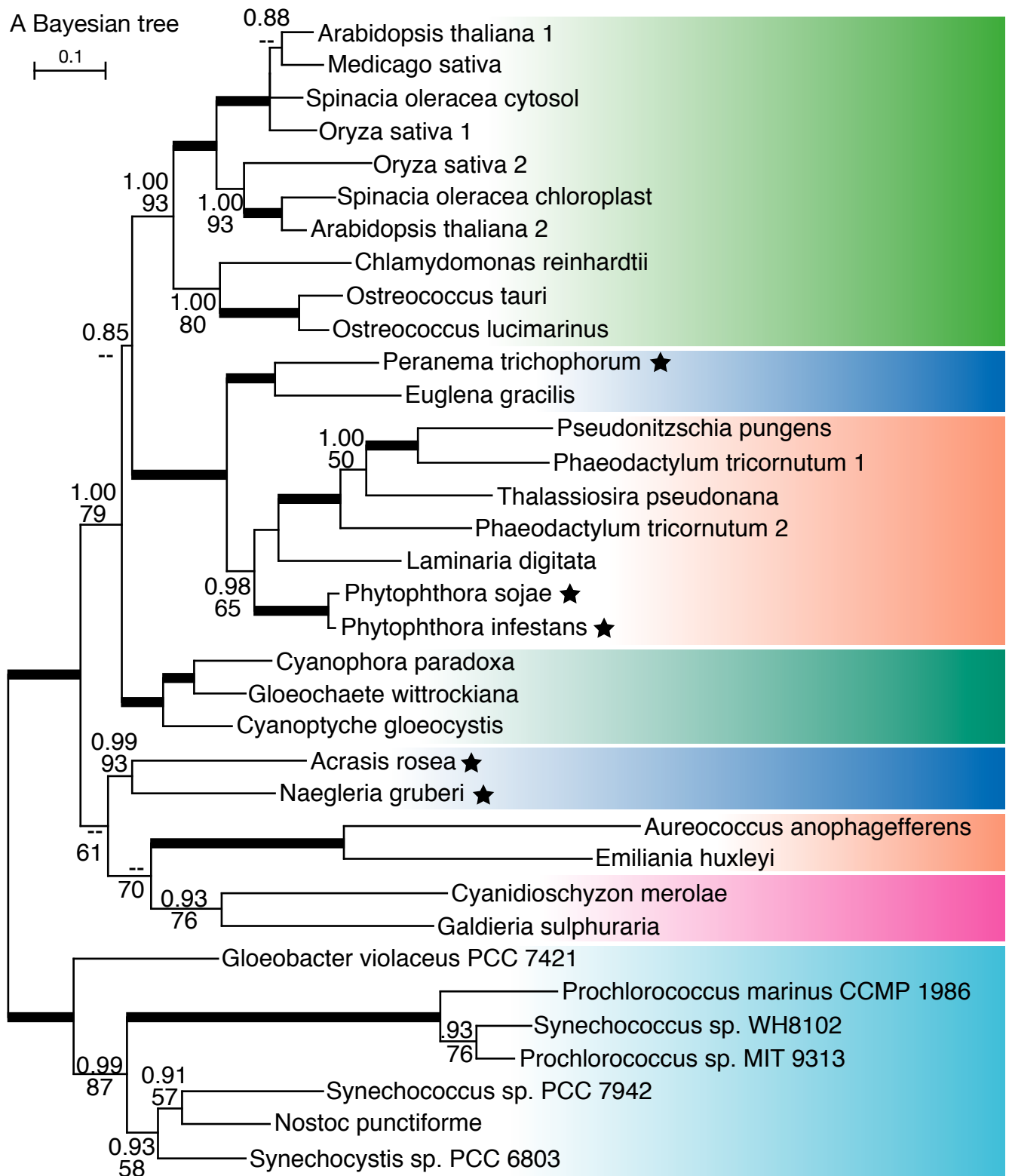

Supplement: Additional file 3 — Supplemental Figure 9. MrBayes consensus tree of 6-phosphogluconate dehydrogenase genes. [file 1471-2148-9-197-S3.pdf]

A Bayesian tree  
BI/ML

0.2

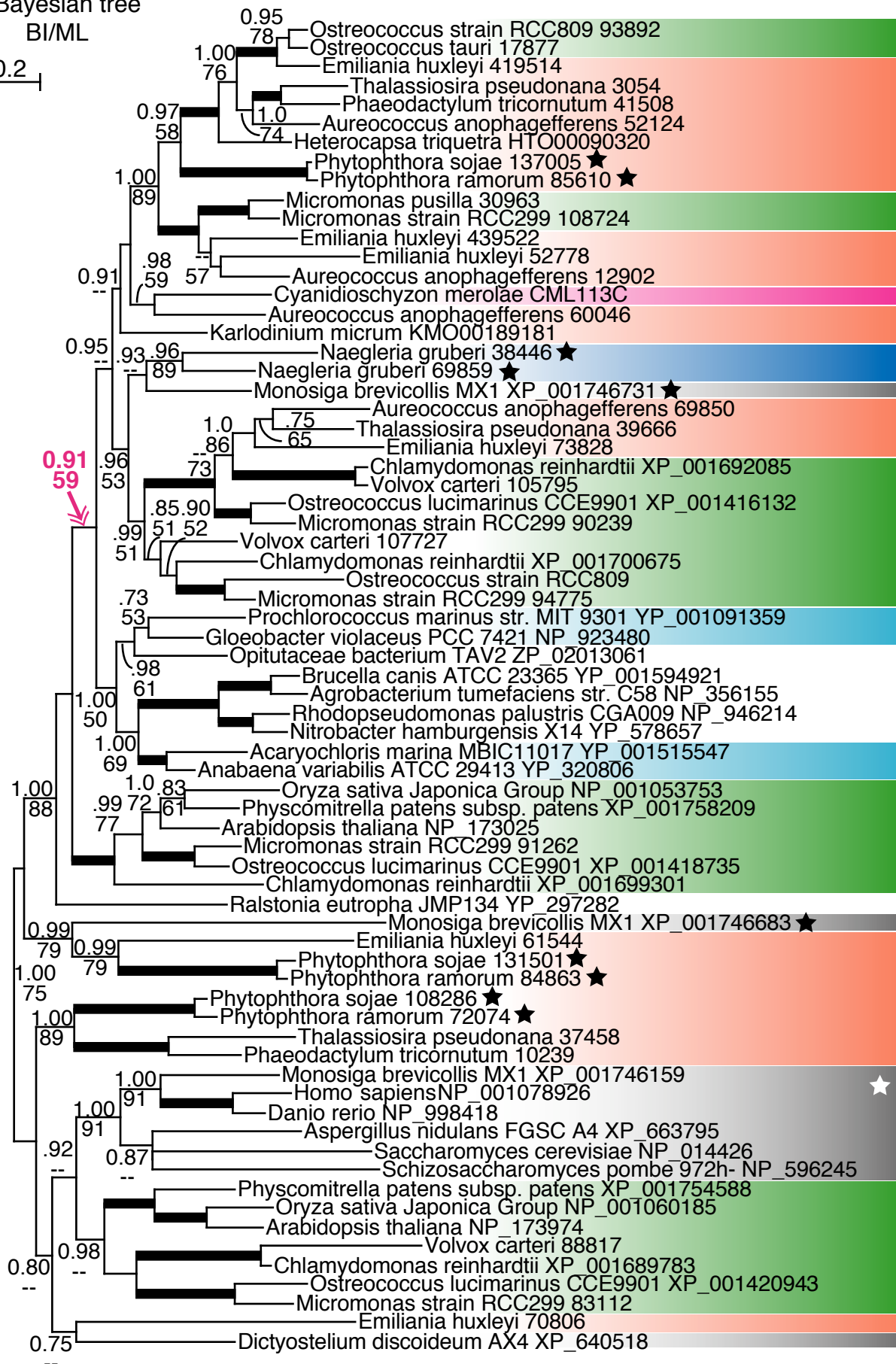

Add\_file 4: Fig. 10 Cobalamin synthesis protein

Supplement: Additional file 4 — Supplemental Figure 10. MrBayes consensus tree of cobalamin synthesis protein genes. [file 1471-2148-9-197-S4.pdf]

# A Bayesian tree

BI/ML

0.2

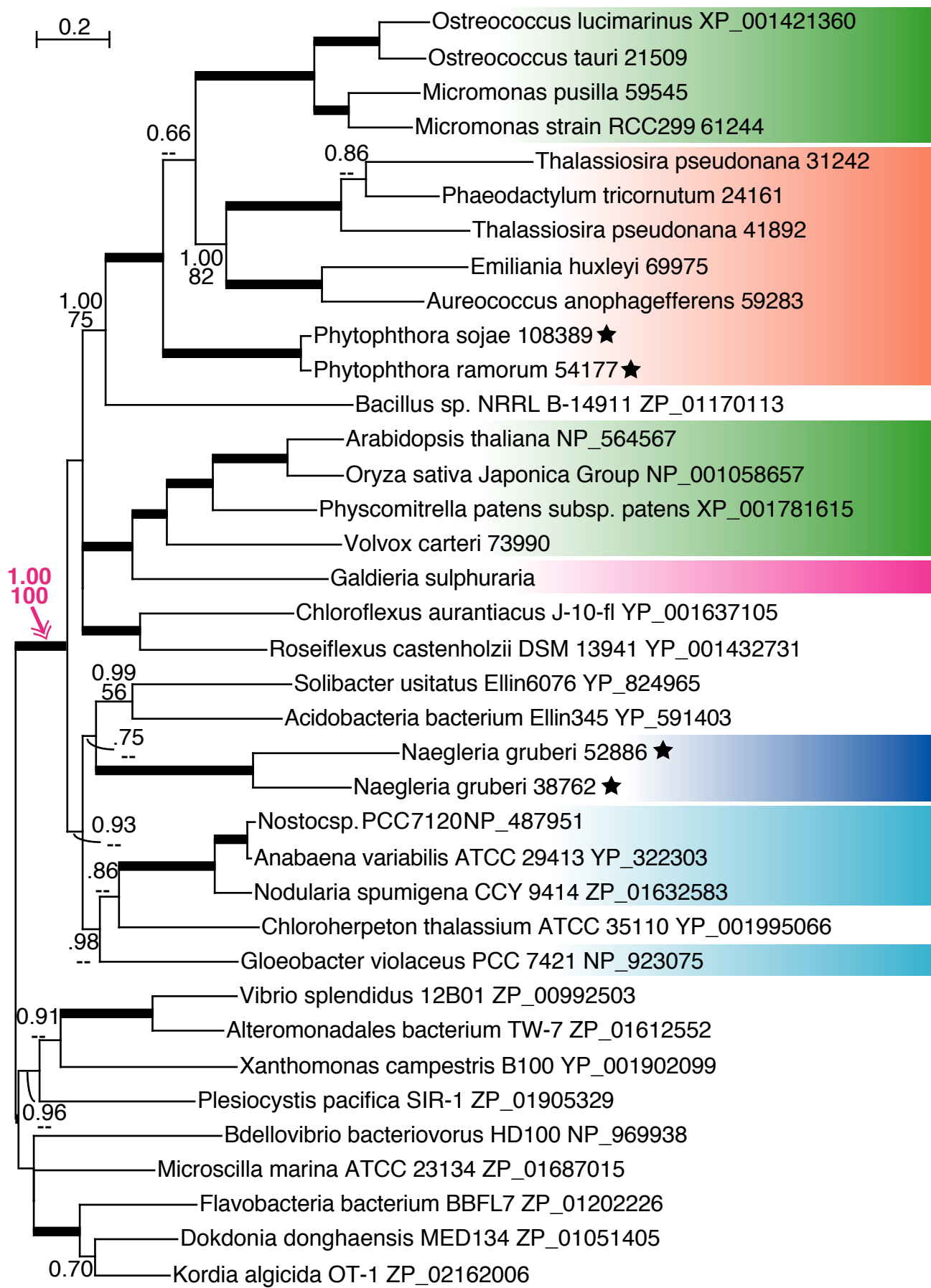

Supplement: Additional file 5 — Supplemental Figure 11. MrBayes consensus tree of oligopeptidase genes. [file 1471-2148-9-197-S5.pdf]

A Bayesian tree

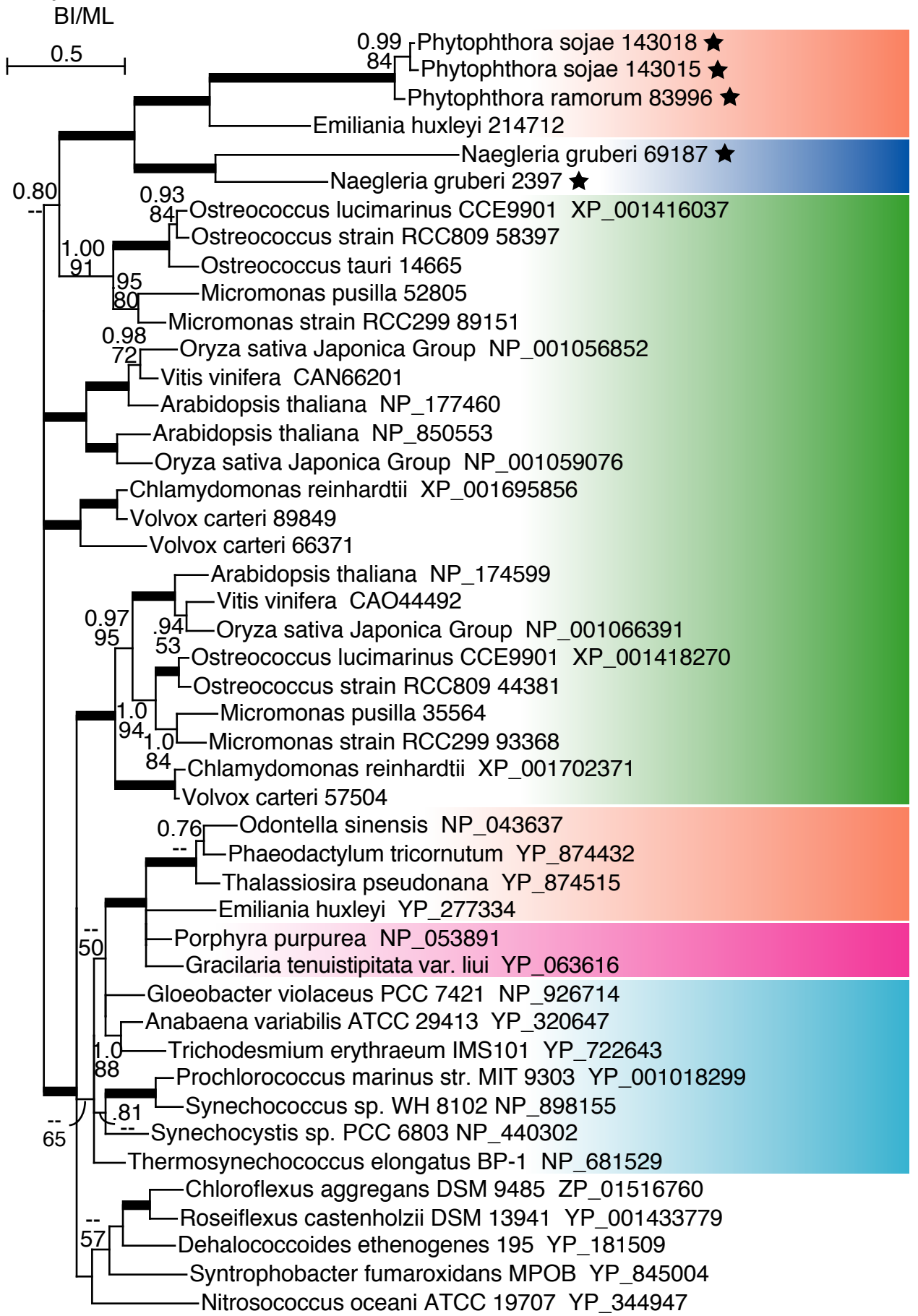

Supplement: Additional file 6 — Supplemental Figure 12. MrBayes consensus tree of YCF45 genes. [file 1471-2148-9-197-S6.pdf]

A Bayesian tree  
BI/ML

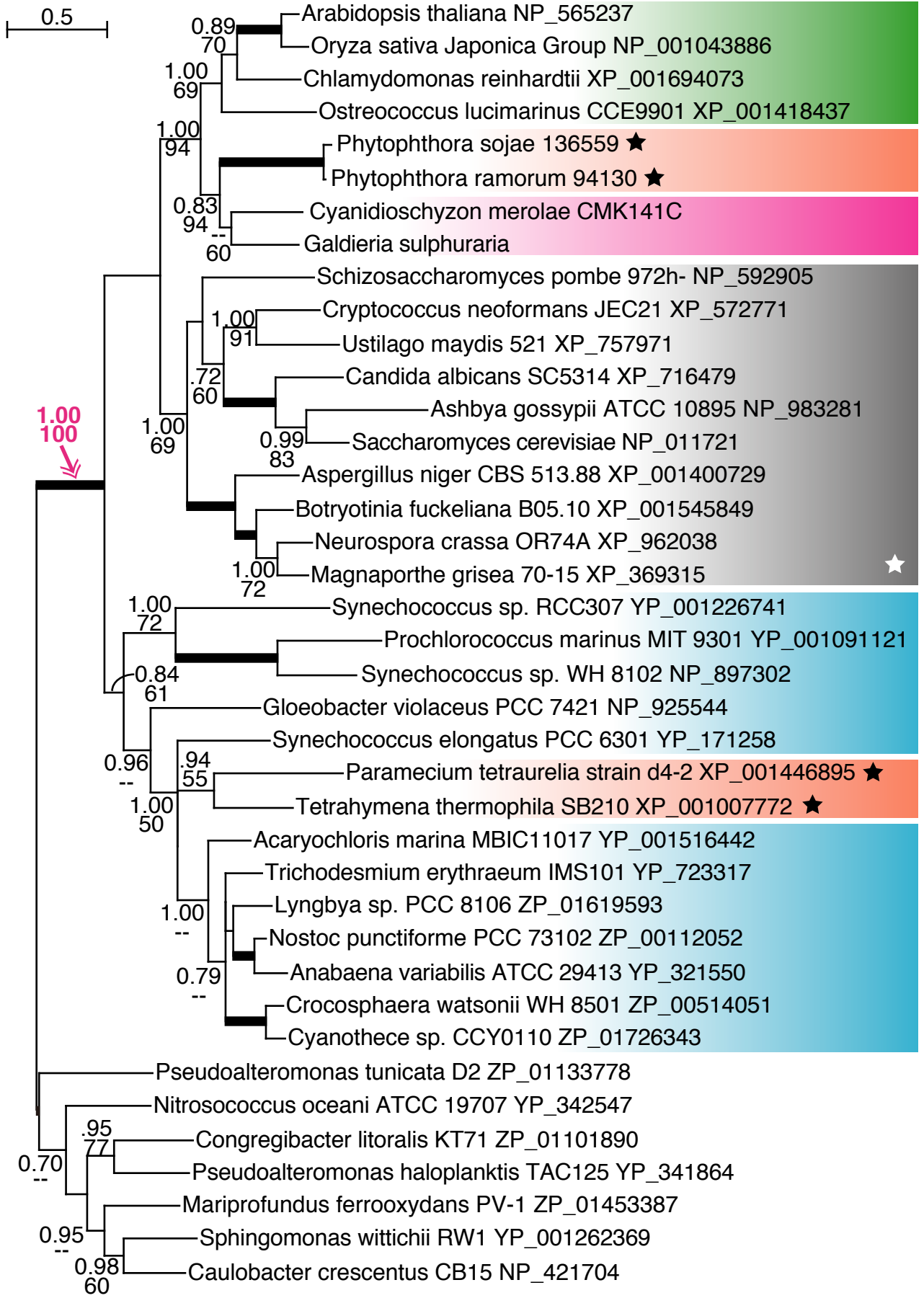

Supplement: Additional file 7 — Supplemental Figure 13. MrBayes consensus tree of glycerate kinase genes. [file 1471-2148-9-197-S7.pdf]

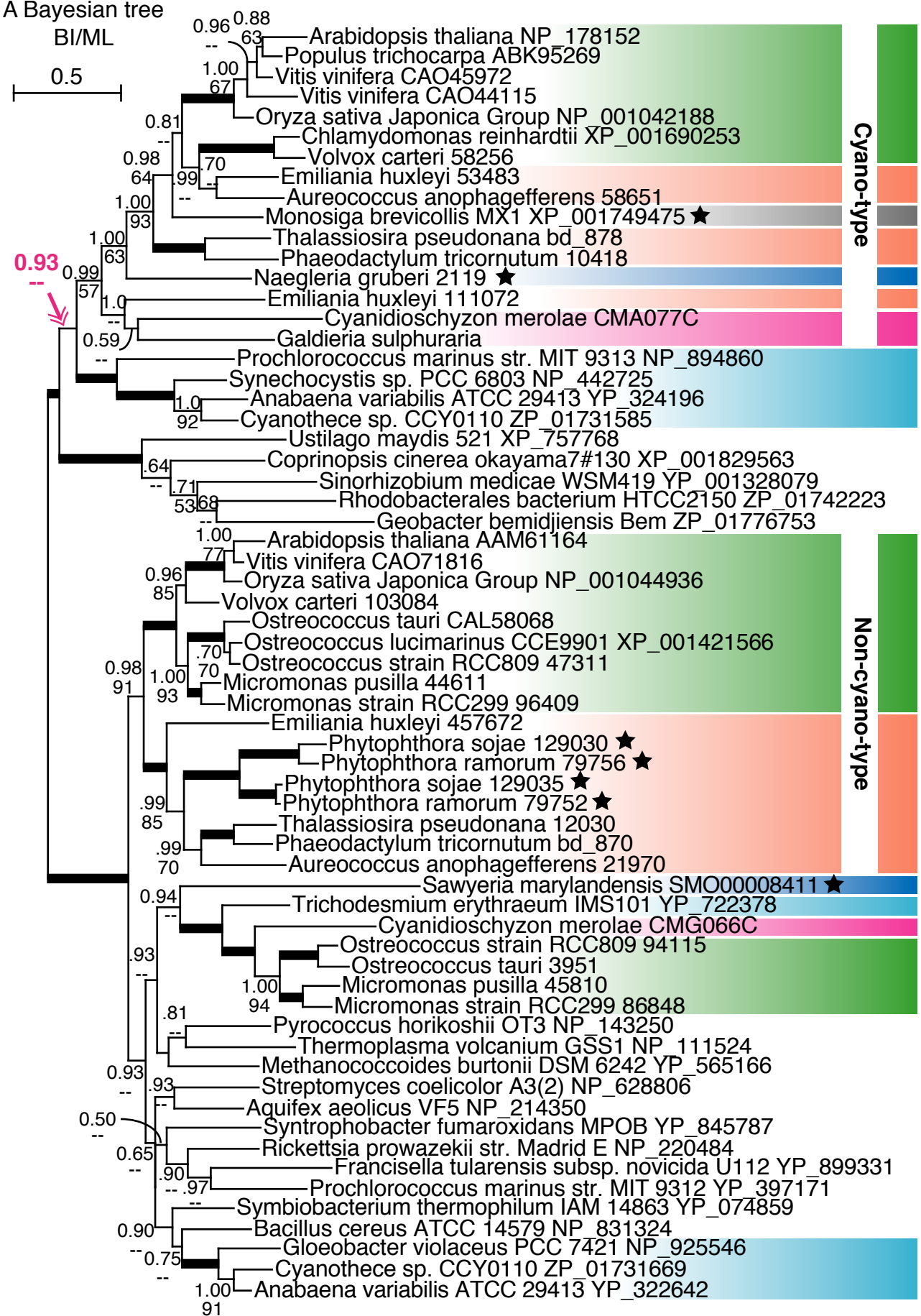

Add\_file 8: Fig. 14 Amino acid aminotransferase

Maruyama et al.

Supplement: Additional file 8 — Supplemental Figure 14. MrBayes consensus tree of amino acid aminotransferase genes. [file 1471-2148-9-197-S8.pdf]

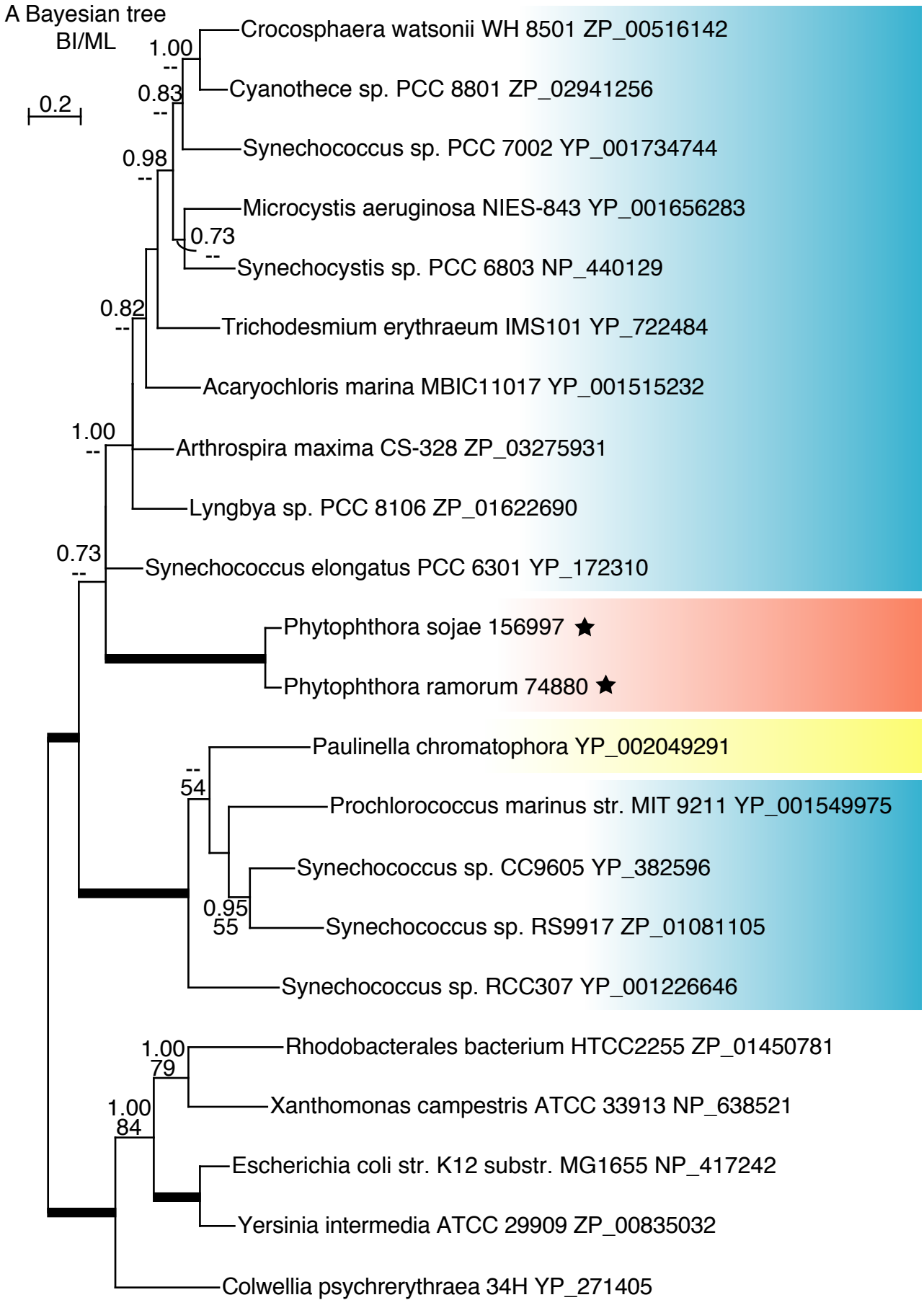

Add\_file 10: Fig. 16 phosphoadenosine phosphosulfate reductase

Supplement: Additional file 10 — Supplemental Figure 16. MrBayes consensus tree of phosphoadenosine phosphosulfate reductase genes. [file 1471-2148-9-197-S10.pdf]

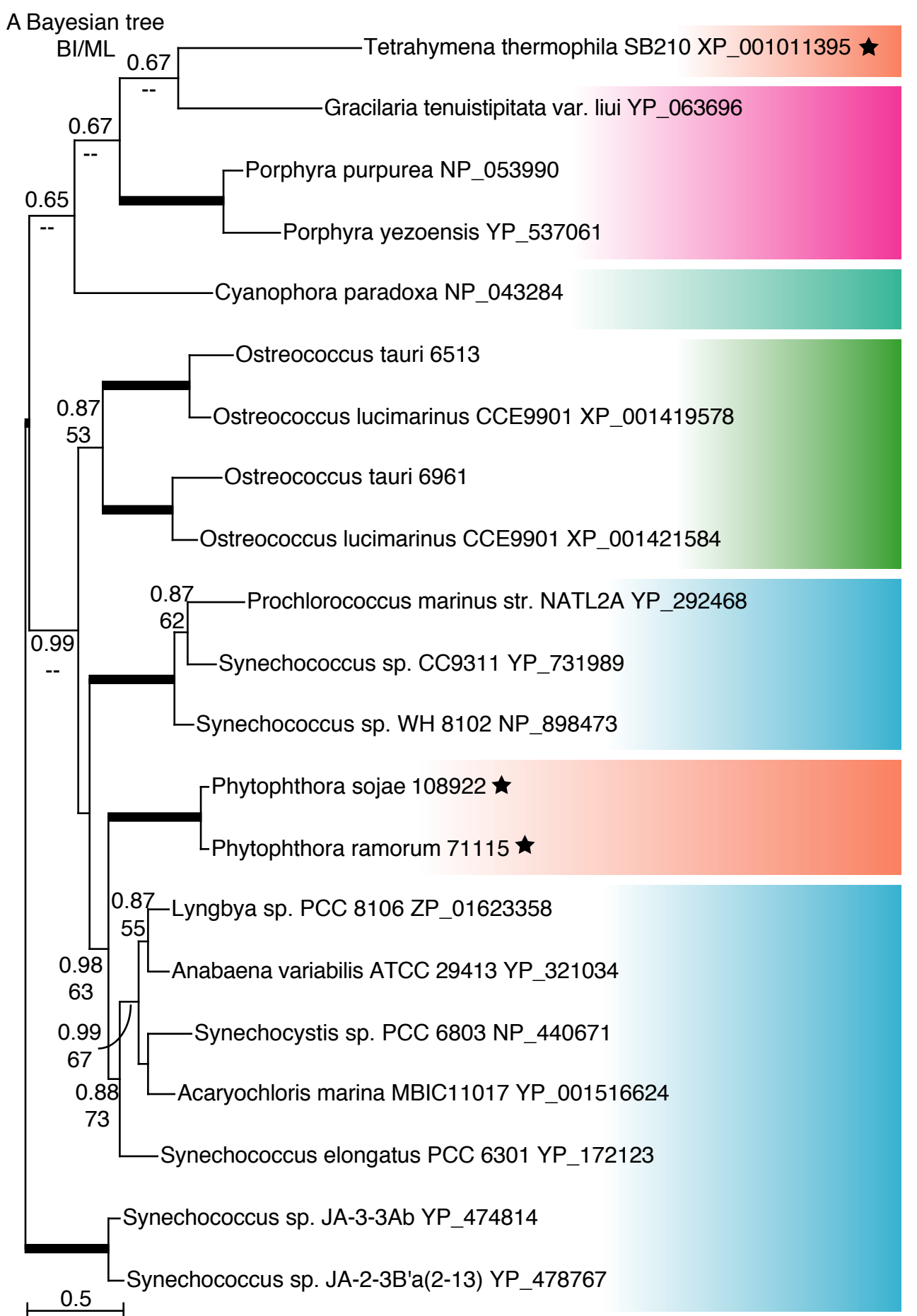

Supplement: Additional file 11 — Supplemental Figure 17. MrBayes consensus tree of YCF21 genes. [file 1471-2148-9-197-S11.pdf]

An ML tree  
BI/ML

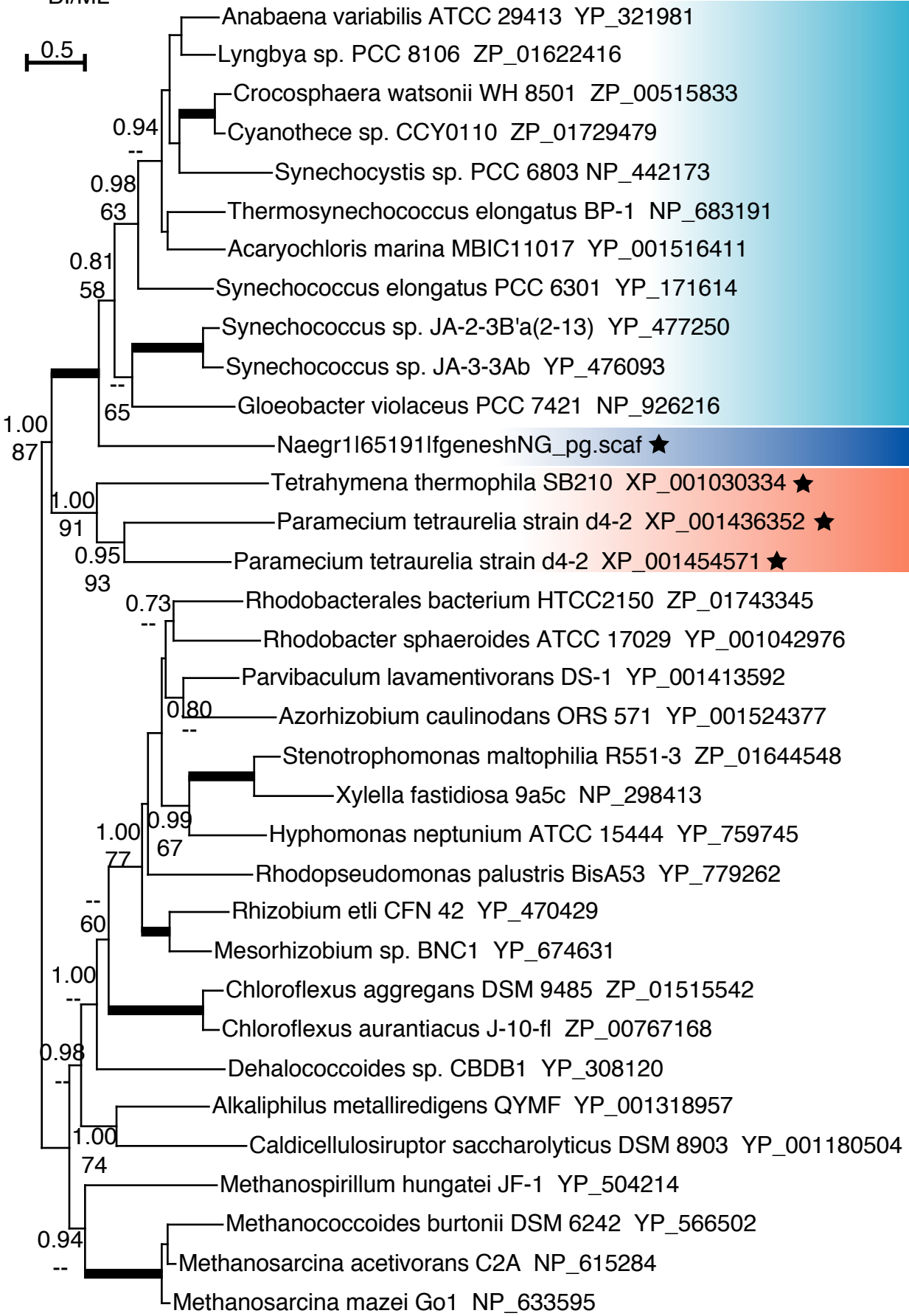

Supplement: Additional file 12 — Supplemental Figure 18. MrBayes consensus tree of hypothetical protein genes. [file 1471-2148-9-197-S12.pdf]
