# Supplementary material for: Cyanobacterial contribution to the genomes of the plastid-lacking protists
Source: BMC Evol Biol. 2009 Aug 11;9:197. doi: 10.1186/1471-2148-9-197 (PMC3087521; doi:10.1186/1471-2148-9-197)
Supplement: Additional file 9 — Supplemental Figure 15. ML consensus tree of glyoxalase I family protein-like genes. [file 1471-2148-9-197-S9.pdf]

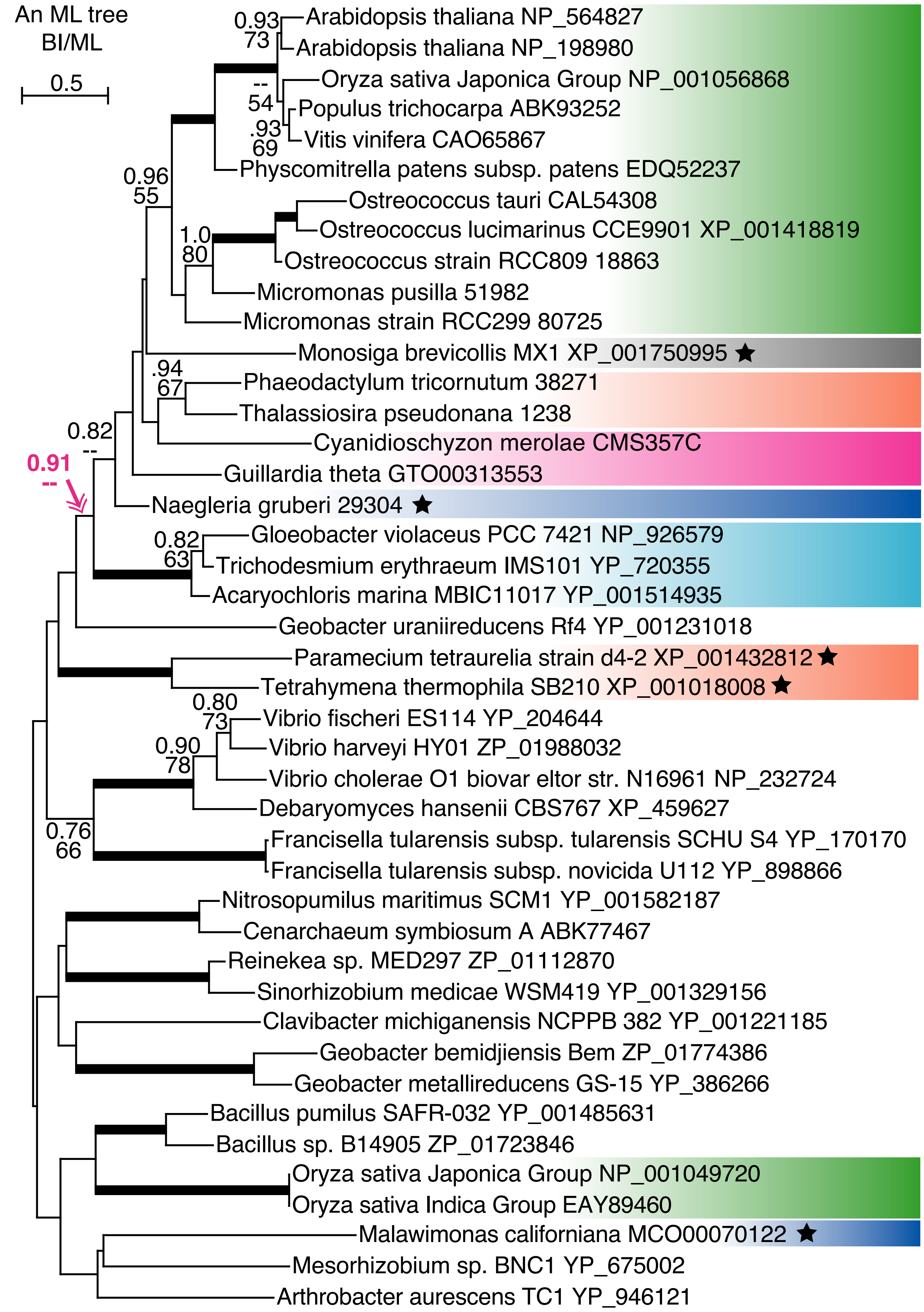

Add_file 9: Fig. 15   Glyoxalase I family protein-like

Maruyama et al.
